# An Integrative Analysis Reveals the Potential Mechanism between Herbal Medicine Yinchen and Immunoregulation in Hepatocellular Carcinoma

**DOI:** 10.1155/2020/8886914

**Published:** 2020-12-29

**Authors:** Zhuomao Mo, Zhirui Cao, Ling Yu, Yongdan Wang, Pan Li, Youwu Lin, Shijun Zhang

**Affiliations:** Department of Traditional Chinese Medicine, The First Affiliated Hospital, Sun Yat-sen University, Guangzhou, Guangdong Province 510080, China

## Abstract

*Aims*. Abundant evidences in traditional Chinese medicine (TCM) supported the therapeutic value of herbal medicine Yinchen in hepatocellular carcinoma (HCC), but the underlying mechanism remains to be investigated. *Main Methods*. The intersection of immune gene set, module genes, HCC-associated genes, and target genes of Yinchen was employed for further analyses. The module genes were identified by weighted gene coexpression network analysis, and the other three gene sets were obtained from public databases. Subsequently, we further explored the clinical value and immunoregulation of the hub gene of intersection. The relevant pathways related to hub gene expression were investigated by gene set enrichment analysis. Finally, the interaction of active compounds and target genes was validated by molecular docking. *Key Findings*. Thirteen active compounds and 90 target genes of Yinchen were included. After constructing the network among Yinchen, target genes, and HCC, BIRC5 was identified as the hub gene. Significant difference was found between the high-expressed group and the low-expressed group in survival and stage. Different immune subtypes also presented significant difference in BIRC5 expression. Moreover, NK cell and T cell (CD4+ effector memory and CD4+ memory resting) were negatively correlated with BIRC5 expression, while CTLA4 and LAG3 were positively correlated. The results of molecular docking further validated a good binding activity of quercetin-BIRC5 interaction. *Significance*. In summary, our research identified for the first time a novel underlying association among herbal medicine Yinchen, BIRC5, immunotherapy, and HCC. We speculated that Yinchen may target the immune checkpoints (CTLA4 and LAG3) and activate the immune cells by suppressing BIRC5.

## 1. Introduction

Hepatocellular carcinoma (HCC) is one of the most common cancers worldwide, which accounts for 85%~90% of all liver cancer cases [1]. Due to the high prevalence and poor prognosis of HCC, it has become the second leading cause of cancer-associated deaths [2]. Partial hepatectomy and liver transplantation are the major treatment for the HCC patients with early stage. But few patients benefit from these treatments because they are mostly diagnosed at an advanced stage. Another treatment for HCC, systemic therapy with sorafenib, is considered as the first-line chemotherapeutic treatment in patients with advanced HCC. Nevertheless, the resistance of sorafenib limits its benefit [3]. Furthermore, immunotherapy has exhibited great potential in HCC, but only a minority of patients can respond to it [4]. Consequently, it is urgent to find the alternative treatment to provide back-up to existing therapies and to improve the prognosis of most HCC patients.

Traditional Chinese medicine (TCM) has been applied for the treatment of HCC for thousand years in China. Based on the specific therapeutic strategies and various herbal medicines, TCM achieved a great success in treating HCC in many studies [5–7]. As the commonly used herbal medicines in liver diseases, Yinchen, Artemisia scoparia in Latin name, is the major herbal medicine in many famous decoctions. Modern pharmacology research demonstrated that Yinchen has the anti-HBV active ingredients [8]. Many decoctions with Yinchen also showed the effect on liver diseases. For example, Yinchen Wuling San decoction presented a favorable outcome in liver cirrhosis from a large cohort study in southwest China [9]. Another study indicated that Yinchen Linggui Zhugan decoction ameliorates nonalcoholic fatty liver disease in rat by regulating the Nrf2/ARE signaling pathway [10]. However, the underlying mechanism between Yinchen and liver disease remains investigation.

A previous study [11] indicated that spleen-deficiency syndrome (SDS) in TCM plays a crucial role in the development of HCC. In the theory of TCM, SDS always induces the “internal dampness” microenvironment of HCC. It is the key point to improve the “internal dampness” microenvironment of HCC patients. Based on the theory of TCM, Yinchen aims at the liver and can ameliorate the “internal dampness.” Meanwhile, it has been reported that the obstruction of immunological recognition function might be caused by “internal dampness” [12]. Consequently, we speculated that Yinchen may have an effect on HCC through the immunologic regulation.

In recent years, systems pharmacology has developed as the prevalent and comprehensive method in herbal medicine research. Systems pharmacology combines bioinformatics and pharmacology to explore the mechanism of action of Chinese herbal medicine [13]. It can construct the drug (active compounds)-targets-disease network and elucidate the complex pharmacological mechanisms of Chinese herbs [14]. In this research, we employed the systems pharmacology analysis to construct the Yinchen-genes-HCC network. We found that the target genes of Yinchen were involved in some immune-associated pathways. Subsequently, we explored the underlying mechanism between hub gene and immunoregulation (immune cell infiltration and immune inhibitors). Our research proposed a novel insight between Yinchen and immunoregulation in HCC, which may help in elucidating the complex mechanism and providing the evidences for experiments.

## 2. Materials and Methods

### 2.1. Data Collection

The compounds and target genes of herbal medicine Yinchen were obtained from the Traditional Chinese Medicine Systems Pharmacology (TCMSP) Database (https://tcmspw.com/tcmsp.php). In terms of HCC targets, the related genes were downloaded from the GeneCards database (https://www.genecards.org/). Meanwhile, the genomic and clinicopathological information of HCC were retrieved from The Cancer Genome Atlas (TCGA) database (https://portal.gdc.cancer.gov/). In addition, the immune-associated genes were obtained from the ImmPort database (https://www.immport.org/home).

### 2.2. Pharmacological Network Construction

The target genes included in our research were selected from four gene sets (the genes from the GeneCards database, the genes from the ImmPort database, the target genes of active compounds of Yinchen, and the genes from the HCC-associated module). Firstly, we identified the active compounds and target genes of Yinchen. The compound is defined as the active compound if oral bioavailability (OB) > 30% and drug‐likeness (DL) > 0.18. The target genes of active compounds were applied to functional pathway analysis through the Kyoto Encyclopedia of Genes and Genomes (KEGG) database. After that, using the gene expression data of TCGA, we performed differential expression analysis to find the differentially expressed genes between tumor and normal samples. The “limma” package under R studio software was employed. Both FDR < 0.05 and ∣log^2^FC | >1 were eligible for further analyses. Then, we performed weighted gene coexpression analysis (WGCNA) to cluster the samples and identify the relevant modules. WGCNA is a systematic biology method which can be used for identifying modules of highly associated genes and associating modules to external traits [15]. Therefore, to identify the interactions between differentially expressed genes, WGCNA was executed to confirm coexpression modules using the topological overlapping measurement, with a module size cutoff ≤ 50 in our research. Meanwhile, the correlation between modules and two clinical traits (stage, grade) was visualized in the heat map. The module which most positively or negatively associated with clinical traits was chosen to find the target genes. The four gene sets were confirmed, and their intersection in the Venn plot was used for further analyses.

### 2.3. Clinical Value and Immunologic Regulation of Hub Gene

The hub gene was retrieved from the intersection of four gene sets. We further explored the clinical value of hub gene in HCC. Firstly, to investigate the time-dependent prognostic value of hub genes, the survival analysis was performed to find the difference between the high and low-expressed groups using the “survival” package. Meanwhile, the association between HCC stage and hub gene expression was investigated. The relation between immune subtype and hub gene expression was explored using the TISIDB website (http://cis.hku.hk/TISIDB/index.php). TISIDB is a web portal for tumor and immune system interaction, which integrates multiple heterogeneous data types [16]. Besides, the underlying correlation between immune cells and hub gene was studied by Tumor Immune Estimation Resource (http://timer.cistrome.org/). It is a comprehensive resource for systematical analysis of immune infiltrates across diverse cancer types and provides different types of immune cells for exploration [17]. Also, to explore the molecular regulatory mechanism between hub gene and immune in HCC, we performed the correlation analysis between hub gene and immune inhibitors by the TISIDB website. A total of 24 immune inhibitors was involved, and the Spearman correlation method was employed. Furthermore, the potential signaling pathways associated with hub gene expression were explored by gene set enrichment analysis (GSEA). GSEA is a computational method which identifies whether a prior defined set of genes shows statistically significant differences between two biological states [18]. The normalized *p* value and enrichment score were used to evaluate the pathways, and the top 10 significant pathways in the high-expressed group were visualized using the “ggplot2” package.

### 2.4. Molecular Docking between Compound and Hub Gene

To further validate the robust association between compound and hub gene, we performed the molecular docking between them. Firstly, the molecular weight and 2D structure of ligand molecular were identified by the PubChem database (https://pubchem.ncbi.nlm.nih.gov/) and the relevant 3D structure of compound was established using ChemOffice software. Meanwhile, the 3D protein structure of hub gene was obtained from the RCSB PDB database (http://www.rcsb.org/). The PyMOL software was used to extract the original ligand conformation of target protein. After the ligand and receptor molecule preparation, we performed the molecular docking. First, we added the polar hydrogen and Gasteiger charges to the presented receptors and ligands with AutoDock Tools. AutoDock Tools was applied to match the hub gene and relevant compound, which employed the Lamarckian genetic algorithm to find the best docking conditions for flexible docking and recorded the docking position. Next, we used the AutoGrid tool to set the parameters of the docking box and adjust the coordinates (*x* = 20.85, *y* = 19.313, and *z* = 12.572) and grid size (*x* = 47.25, *y* = 47.25, and *z* = 47.25). The binding energy was used to evaluate the result of molecular docking. The scoring function of AutoDock stipulates that when score < 0, it means that the combination of target and active compound can bind stably, and the greater the absolute value, the stronger the stability [19]. Finally, we applied the PyMOL software to analyze and visualize the docking results of compounds and proteins.

## 3. Results

### 3.1. Identification of Hub Gene

**Figure 1:**
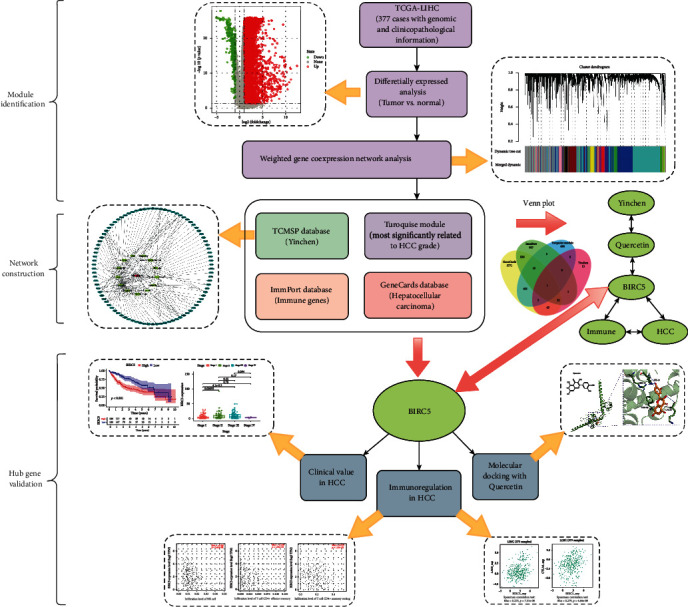
The flowchart of this study.

First of all, we summarized the flowchart and showed it in Figure 1.

The clinical details of the patients from TCGA employed in our study are summarized in Table 1.

**Figure 2:**
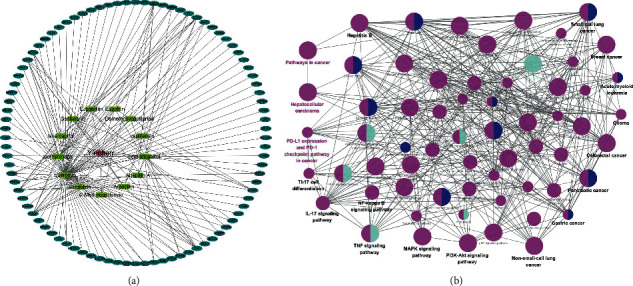
The Yinchen-active compounds-target genes network and KEGG signaling pathways: (a) showed the Yinchen-active compounds-target genes network; (b) showed the KEGG signaling pathways.

As illustrated in Figure 2(a), the network which included Yinchen, 13 active compounds, and 90 target genes was generated.  Figure 2(b) shows the significant signaling pathways from 90 target genes. The target genes referred to the various functional pathways, including the pathways related to many cancers. Particularly, we observed that some important immunologic pathways, such as PD-L1 expression and PD-1 checkpoint pathway in cancer and Th17 cell differentiation and IL-17 signaling pathway, were significantly associated with target genes.

**Figure 3:**
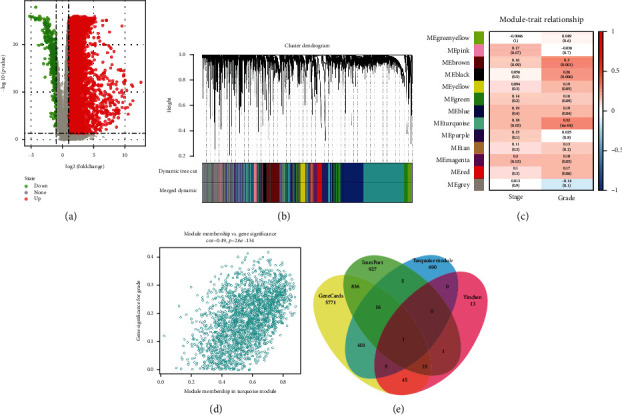
Identification of module and hub gene: (a) showed the volcano plot of differentially expressed analysis, (b) showed the cluster plot, (c) showed the correlation between modules and clinical parameters, (d) showed the correlation between genes and age, and (e) showed the Venn plot.

Concerning the gene set associated with TCGA, we firstly performed the differential expression analysis and found that 7667 differential expressed genes were included in subsequent analysis (Figure 3(a)). The results of WGCNA in Figure 3(b) showed the clustering samples and corresponding modules. We also found the strongest association between turquoise module and HCC grade (Figures 3(c) and 3(d)). Consequently, we considered that the genes in the turquoise module was important for HCC and employed them for further analyses. The Venn plot in Figure 3(e) showed the intersection of four gene sets. Only one gene (BIRC5) was included in the intersection.

### 3.2. Clinical Value and Immunoregulation of BIRC5

**Figure 4:**
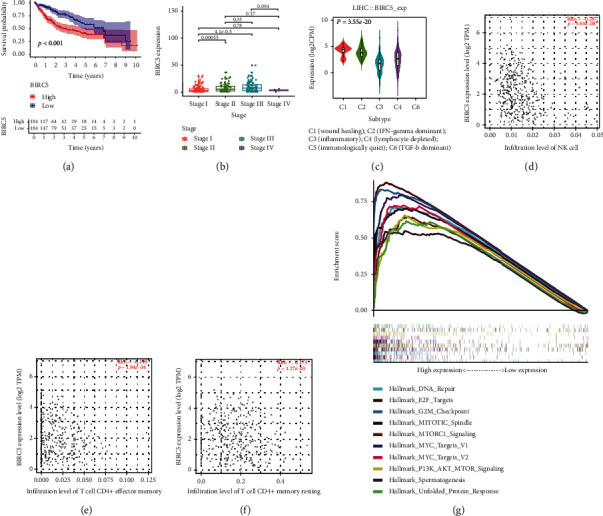
The correlation between clinical traits and BIRC5, immune and BIRC5: (a) showed the survival analysis results, (b) showed the correlation between stage and BIRC5 expression, (c) showed the correlation between immune subtypes and BIRC5 expression, (d–f) showed the correlation between immune cells and BIRC5 expression, and (g) showed the significant pathways enriched in the high-expression group.

As shown in Figure 4(a), the high-expressed group of BIRC5 presented the worse prognosis than the low-expressed group in HCC. Comparing with stage I, the patients in stage II and stage III showed the higher expression of BIRC5 (Figure 4(b)). Besides, the significant difference of BIRC5 expression was found in different immunological subtypes, in which inflammatory subtype presented the lowest BIRC5 expression. We further explored the underlying association between BIRC5 and immune cells. The results in Figures 4(d)–4(f) demonstrated that BIRC5 expression was significantly associated with three types of immune cell, including NK cell, T cell CD4 effector memory, and T cells CD4 memory resting. Simultaneously, the high expression of BIRC5 significantly enriched in various signaling pathways, including DNA repair, E2F targets, G2M checkpoint, mitotic spindle, MTORC1 signaling, Myc targets V1, Myc targets V2, PI3K AKT MTOR signaling, spermatogenesis, and unfolded protein response.

**Figure 5:**
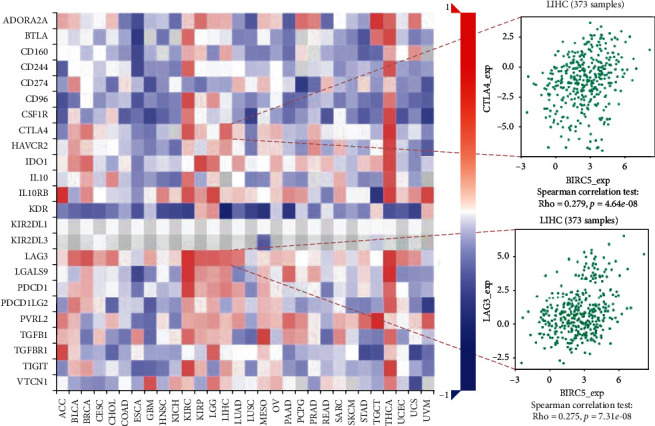
The correlation between immune inhibitors and BIRC5 expression.

Furthermore, we explored the potential relation between BIRC5 expression and immune inhibitors and found that CTLA4 and LAG3 were more strongly associated with BIRC5, respectively (Figure 5).

### 3.3. Validation of Quercetin-BIRC5 Interaction by Molecular Docking

**Figure 6:**
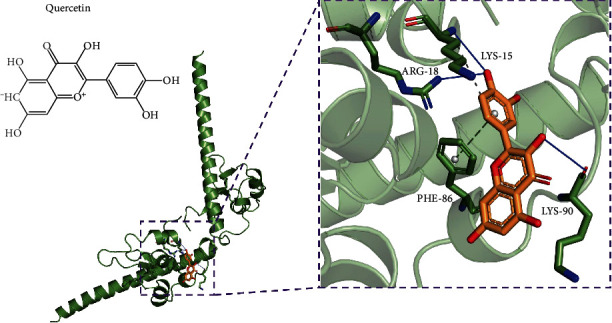
The molecular docking between BIRC5 and quercetin.

To further verify the binding capacity between active compound and hub gene, molecular docking analysis was performed. Only one hub gene (BIRC5) and its corresponding active compound (quercetin) were employed in molecular docking analysis. The PDB code of BIRC5 is “6SHO,” and it can be referred from the previously published study [20]. The binding energy of quercetin-BIRC5 interaction was -7.5, which indicated that they possessed good binding activity. The complex quercetin-BIRC5 was stabilized by hydrogen bonds with residues including Lys15, Arg18, and Lys19, respectively. The results in Figure 6 also showed the pi-stacking interaction between Phe86 (residues of BIRC5) and quercetin. Additionally, it has been found the hydrophobic interaction between Lys15 and quercetin.

## 4. Discussion

In the theory of TCM, HCC can be referred to as the disease “abdominal dropsy,” which is mainly caused by “external dampness” and “internal dampness.” Therefore, the therapeutic strategy for HCC in TCM is to resolve the “dampness.” At the same time, the “dampness” in TCM has been verified to be correlated with the obstruction of immunological recognition function [12]. As one of the representative herbal medicines in resolving dampness, Yinchen has been used in liver diseases in TCM for many years. However, the underlying mechanism between Yinchen and HCC is unclear. In this research, we speculated the active compounds of Yinchen have an effect on HCC by immunoregulation.

The core of systems pharmacology is to construct the drug (active compounds)-target genes-disease network. Firstly, we established a network involving Yinchen, active compounds, and target genes. Subsequently, we identified the genes associated with HCC and immune-associated genes and finally confirmed that BIRC5 was the hub gene in the network. BIRC5, baculoviral IAP repeat containing 5, is a 16.5 kDa intracellular protein that belongs to the inhibitor of the apoptosis protein family [21]. It interacts with the mitotic spindle apparatus to regulate cell division and modulate the function of effector cell death proteases resulting in inhibition of apoptosis [22, 23]. It has been reported that BIRC5 plays a crucial role in many cancers. Dimitrov-Markov et al.'s study [24] indicated that BIRC5 is the new target to control metastasis in pancreatic cancer. Another study [25] suggested BIRC5 as a putative predictive biomarker in squamous cell lung carcinomas with TP53 mutation. Meanwhile, various researches [26–28] revealed that BIRC5 expression significantly correlated with poor prognosis, aberrant methylation, and immune cell infiltration in HCC. Our research also found that higher expression of BIRC5 leads to the poor overall survival and advanced stage, which verified the importance of BIRC5 in HCC.

In our research, quercetin is the unique active compound targeted BIRC5. Quercetin (3,3′,4′,5,7-pentahydroxyflavone) is a compound that belongs to the flavonoid family, which was abundant in the form of glycoside in a variety of plants. Over the past years, both in vivo and in vitro experiments have revealed that quercetin can present antitumor effects by altering cell cycle progression, inhibiting angiogenesis and metastasis progression, and affecting autophagy [29]. Concerning HCC, it has been verified that quercetin inhibited the growth factor-induced migration of HCC cells by suppressing the AKT pathway [30]. Another study [31] also demonstrated that quercetin can inhibit HCC progression which partly correlated with the JAK2/STAT3 pathway. Simultaneously, we further validated the interaction between quercetin and BIRC5 by molecular docking. The previous study also reported that quercetin effectively inhibited human HCC cell proliferation and induced apoptosis by downregulating the expression of BIRC5 in vitro [32]. Since BIRC5 and quercetin showed the momentous value in HCC, it is necessary to investigate the underlying association between quercetin-BIRC5 interaction and HCC.

Interestingly, we observed that the target genes of Yinchen were involved in some immunologic pathways, which verified the underlying association between the immune system and Yinchen. Based on the theory of TCM, Yinchen can improve the microenvironment of spleen deficiency by resolving dampness. Since the spleen was associated with immunity in TCM, we speculated that Yinchen may have an effect on HCC by modulating immune-associated factors. As expected, we found BIRC5 was significantly associated with different immune cells and immune inhibitors. Particularly, BIRC5 was positively correlated with CTLA4 and LAG3. CTLA4 (cytotoxic T-lymphocyte-associated protein 4) is constitutively expressed in T cells and attenuates immune responses when bound to CD86 or CD80 on the surface of antigen-presenting cells [33]. LAG3 (lymphocyte activation gene-3), also named CD223, widely expressed on CD4+ T cells, CD8+ T cells, and NK cells [34]. High expression of LAG3 is correlated with T cell dysfunction in tumor environment [35]. Both CTLA4 and LAG3 have been considered as the immune checkpoint targets in cancer immunotherapy [36]. Meanwhile, CD4+ T cell and NK cell were negatively related to BIRC5 expression in our results. Consequently, we supposed that Yinchen may target the immune checkpoints (CTLA4 and LAG3) and activate the immune cells by suppressing BIRC5 expression. Moreover, we found that BIRC5 expression was significantly correlated with different immune subtypes and C3 subtype (inflammatory) presented the lowest BIRC5 expression. C3 subtype was defined by elevated Th17 and Th1 genes and presented low to moderate tumor cell proliferation [37]. A previous study indicated that C3 subtype presented the most favorable prognosis may attribute to display a type I immune response in cancer control [37]. Therefore, further investigations exploring whether lower BIRC5 expression presented the favorable prognosis by type I immune response are needed.

To our knowledge, it is the first research to construct the Yinchen-quercetin-BIRC5-HCC network and investigate the immunologic mechanism among them. Differing from the common network pharmacology, the results of WGCAN were considered into the intersection of gene sets, which make the hub gene more reliable. Also, combination of TCM and immunity is a novel insight in shedding light on the complex mechanism of TCM. Furthermore, we proposed the potential interaction, Yinchen (quercetin)-BIRC5-immune checkpoints (CTLA4, LAG3)-HCC, which may help in the validation of functional experiments. Nevertheless, some limitations in our research have to pinpoint. First, only one herbal medicine (Yinchen) was explored in our research, but the formulas including various herbal medicines were prevalently used in clinic. We considered that employing the network pharmacology in formula was the intrinsic weakness, because it is hard to determine the major effective herbal medicine and the major effective compound when multiple herbal medicines were used in formula. Also, it is hard to identify the synergistic effect among herbal medicines. We are looking forward to more novel and comprehensive methods to explore the complex mechanism of formula. Second, the pathway results of GSEA were preliminary; it is necessary to perform more functional experiments to validate.

## 5. Conclusion

In summary, our research identified for the first time a novel underlying association among herbal medicine Yinchen, BIRC5, immunotherapy, and HCC. We speculated that Yinchen may target the immune checkpoints (CTLA4 and LAG3) and activate the immune cells by suppressing BIRC5. The insights in our research may help in elucidating the complex mechanism of TCM and further experimental validation.

## Figures and Tables

**Table 1:** Baseline patient characteristics in TCGA cohorts.

Clinical characteristics		Number	Percent (%)
TCGA-LIHC (*n* = 377)			
Survival status	Survival	249	66
	Death	128	34

Age (1 patient missing)	≤65 years	235	62.5
	>65 years	141	37.5

Gender	Female	122	68
	Male	255	32

Stage (24 patients missing)	I	175	50
	II	87	24.6
	III	86	24.4
	IV	5	1

Grade (5 patients missing)	G1	55	14
	G2	180	48
	G3	124	33
	G4	13	5

T classification (3 patients missing)	T1	185	49
	T2	95	26
	T3	81	22
	T4	13	3

## Data Availability

The data used to support the findings of this study are included within the article.
